# Editorial: Exploring digital mental health solutions for domestic violence victims in the post-pandemic era

**DOI:** 10.3389/fpubh.2026.1816776

**Published:** 2026-03-18

**Authors:** Zhaohui Su, Chaojun Tong, Bindi Bennett, Wissam El-Hage, Dean McDonnell, Claudimar Pereira da Veiga, Yu-Tao Xiang

**Affiliations:** 1 School of Public Health, Southeast University, Nanjing, China; 2 Key Laboratory of Environmental Medicine Engineering, Ministry of Education, School of Public Health, Southeast University, Nanjing, China; 3 National Centre for Reconciliation, Truth, and Justice, Federation University Australia, Ballarat, VIC, Australia; 4 The Clinical Investigation Centre, Université de Tours, Tours, France; 5 Department of Humanities, South East Technological University, Carlow, Ireland; 6 Fundação Dom Cabral - FDC, Nova Lima, MG, Brazil; 7 Unit of Psychiatry, Department of Public Health and Medicinal Administration, Faculty of Health Sciences, Centre for Cognitive and Brain Sciences, University of Macau, Taipa, Macao SAR, China

**Keywords:** artificial intelligence, domestic violence, ethics, mental health, public health economics hospital, social justice, technological interventions, women's health

Mental health is a global priority, particularly in the post-pandemic era, where substantial psychological distress has been observed across sectors, from healthcare professionals to vulnerable communities (1–3). Domestic violence victims exemplify these challenges. The coronavirus disease 2019 (COVID-19) pandemic not only magnified the global burden of domestic violence but also disrupted access to traditional mental health and social support services. In response, digital mental health solutions have emerged as critical alternatives for providing timely, scalable, and potentially personalized care to survivors (4, 5). This collection of research, under the Topic entitled “*Exploring digital mental health solutions for domestic violence victims in the post-pandemic era*,” explores the post-pandemic landscape of digital interventions for domestic violence victims, spanning advanced artificial intelligence (AI) frameworks, hybrid care models, epidemiological analyses, and systematic reviews. Together, these studies illuminate both the promise of digital innovation and the persistent challenges related to effectiveness, accessibility, ethics, and long-term implementation. By integrating technological, clinical, and public health perspectives, the Research Topic offers a comprehensive understanding of how digital tools can be effectively and responsibly leveraged to support survivors in the post-COVID era.

Domestic violence is not a new phenomenon, yet the modern world presents uniquely dangerous circumstances. First, perpetrators now have access to unprecedented tools—from tracking devices that can be exploited for stalking to AI-generated deepfake “revenge pornography”—that can inflict rapid and widespread harm. Second, weak or absent regulatory safeguards persist, as laws and policies often fail to keep pace with emerging technologies. Third, effective public health interventions remain scarce, with many programs confined to research settings or underfunded and unable to scale. During the COVID-19 pandemic, lockdown measures further exacerbated domestic violence by trapping individuals in unsafe environments and weakening social safety nets. Yet the same technological forces that can worsen abuse also hold potential for prevention and support. This Research Topic highlights that innovation, informed by the lessons of the pandemic, offers an opportunity to re-examine domestic violence through new, multidimensional lenses.

## Technological innovations and personalization of care

A main theme of this Research Topic is the use of emerging technologies to personalize and enhance mental health support for survivors. Zhang advances this effort through a multimodal, AI-driven framework that models dynamic psychological trajectories using behavioral, physiological, and narrative data. By embedding interpretability and ethical safeguards, this work addresses longstanding concerns about transparency, bias, and safety in AI-based mental health systems. Complementing this, Feng emphasizes the value of contextual intelligence, identifying risk and protective factors—such as controlling behaviors, alcohol use, fear of the partner, and family violence histories—that can inform risk-sensitive digital tools. Collectively, these studies argue that effective digital mental health solutions must evolve beyond static symptom monitoring toward adaptive, context-aware, and predictive models.

## Effectiveness of interventions

Despite the rapid pace of technological development, questions about real-world effectiveness persist. Yu et al. systematically review post-COVID technology-based interventions and report mixed outcomes: some digital tools produce measurable mental health benefits, while others show limited or no effect. The study reveals that methodological inconsistencies, small sample sizes, and practical barriers—such as digital literacy and sustained engagement—continue to limit the generalizability of results (Yu et al.). One promising direction lies in hybrid models that combine digital delivery with in-person or embodied care. Machorrinho et al. describe a hybrid psychomotor intervention implemented in shelter homes to ensure continuity of care during relocation and instability. Their work underscores the importance of implementation science, ensuring that digital mental health programs are feasible, sustainable, and embedded within existing community support systems rather than operating in isolation.

## Life course trauma and public health solutions

This Research Topic also positions digital mental health within a life-course and public health framework. Chen et al. demonstrate the lasting mental health effects of adverse childhood experiences, identifying trauma profiles closely linked to adult anxiety and depression. These findings highlight the cumulative and intergenerational nature of violence-related trauma and reinforce the need for interventions that address long-term vulnerabilities instead of focusing solely on acute crises. Properly designed digital platforms, with capacities for longitudinal monitoring and early intervention, are especially well-suited to such trauma-informed approaches.

## Ethics, access, and equity in digital mental health

Ethical and equity considerations emerge as central concerns across all contributions. Survivors of domestic violence often face heightened risks related to data privacy, surveillance, algorithmic bias, and unequal access to technology. Without careful governance, digital interventions may inadvertently exacerbate harm. This Research Topic emphasizes the need for survivor-centered design, transparent ethical frameworks, and empathetic policies that make digital innovations accessible, culturally sensitive, and safe for those most at risk.

## Conclusion

The studies in this Research Topic reveal both the transformative potential and the persistent challenges of digital mental health solutions for domestic violence survivors in the post-pandemic world. Advances in AI, hybrid care models, and digital interventions offer new opportunities for timely, personalized, and scalable support. Yet realizing these benefits requires rigorous evaluation, interdisciplinary collaboration, and constant attention to accessibility, equity, and survivor safety. Moving forward, a holistic and trauma-informed research agenda—linking technological innovation with public health insight, clinical practice, and policy—will be essential to ensuring that digital mental health solutions genuinely and responsibly improve outcomes for domestic violence survivors in an increasingly digital world. The question is no longer whether “to act, or not to act”; rather, it is how to act swiftly, intelligently, and compassionately.
